# Herbal cosmetics in ancient India

**Published:** 2008-10

**Authors:** Kunda B. Patkar

## INTRODUCTION

The ancient science of cosmetology is believed to have originated in Egypt and India, but the earliest records of cosmetic substances and their application dates back to Circa 2500 and 1550 B.C, to the Indus valley civilization.[1] There is evidence of highly advanced ideas of self beautification and a large array of various cosmetic usages both by men and women, in ancient India. Many of these practices were subtly interwoven with the seasons (Sanskrit: *Rutus*) and the normal rituals of life (Sanskrit: *Dinacharyā*). Significantly, the use of cosmetics was directed not only towards developing an outwardly pleasant and attractive personality, but towards achieving merit (Sanskrit: *Punya*), Longevity with good health (Sanskrit: *Aayush* and *Aarogyam*) and happiness (Sanskrit: *Anandam*). In this context, the earliest reference of a beautician is from the great epic Mahabharata, where the Pandavas were in exile incognito. Draupadi worked for the queen of Virāta (Northern district of India). She called herself Sairandhri (A female attendant in the women's sections of the palace). There is a reference of her carrying a Prasādhana Petikā (A vanity case containing substances to beautify, toiletries and accessories to decorate).[2,3]

The word cosmetics defined as “Substances of diverse origin, scientifically compounded and used to i) cleanse, ii) allay skin troubles, iii) cover up imperfections and iv) beautify” (Encyclopedia Britannica, 1970), is used in this paper in a wider sense to include Oral hygiene as well.

Different *Lepās* (Masks or applications) were recommended for different seasons for body beautification. The ingredients used during the cold seasons were quite different from those used in warm seasons. In fact *Ashtānga Hridaya* (a 1500 year old book of Ayurveda) offers six different formulations to be used for the six seasons of the year. Similarly special cosmetic *Tailams* (Oils) and *Ghritas* (Clarified butter or ghee) were used for facial beautification. Superfluous hair was considered to be a stigma and a large number of depilatory agents were recommended to get rid of it. Special ingredients were used for hair washes. Many remedies have been indicated for hair growth, prevention of falling hair and premature graying. Hair dyes, fragrant hair rinses and fumigants were also in use. Fragrant bath powders and body deodorants also find frequent mention. Oral hygiene in the form of care of teeth, mouth deodorants and coloring of lips were daily chores to be religiously pursued. It appears that the whole range of modern cosmetic usage was conceived by the ancient Indians and was practiced with the help of natural resources then available.

In the book published by the author,[4] 210 different botanicals have been studied and 314 formulations are listed and described. Of these 151 botanicals are identified, 21 are unidentified, and 38 remain uncertain. The scientific name of the plant species is followed by references equating the Sanskrit/Prakrit name or synonym and the references given in parenthesis relate to the scientific name only.

A few examples are cited below to understand the trouble taken by ancient researchers to evolve the Science of cosmetics.

## RAJA SERFOJI'S EXPERIMENTS IN COSMETICS

Raja Serfoji ascended the throne of Tanjore (Thanjavur in Tamil Nadu State South India) in 1788 A.D and ruled till 1832 A.D. He is the architect of the great library called “Serfoji's Saraswati Mahāl,” at Tanjore. The Raja was very interested in medical preparations and research. He tested many recipes by actually having them administered to patients and had case histories of patients recorded by British doctors. He established an Institute of Medical Research called the “Dhanvantari Mahāl”, where experiments were conducted, and he selected a few thousand efficacious recipes after actually testing them. They were then given to Tamil pandits to be woven into verse and written on palm leaves or paper. The Tamil versions were in turn translated into colloquial Marathi (known as “Bakhar Marathi” or Old spoken and written Marathi during 18^th^-19^th^ Century A.D.) for the benefit of Marathi speaking people. These recipes are called *Anubhoga Vaidya Bhāga*, which means recipes tested by experience. Several of these manuscripts have now been published along with a prose version.[5] Many ancient families of Tanjore still possess medicines prepared in the Dhanvantari Mahāl, bearing the original seals indicative of the sample and the date of its preparation.[5]

To facilitate the preparation of medicines, Raja Serfoji established a grand herbarium in his palace where he had a nursery which supplied plants for experiments, to the Dhanvantari Mahāl. He also used artists and illustrated these plants in water color paintings and bound them in books for quick future reference. Few of his formulas are given below:

### Lip balm / lip salve

“Cracked lips, besides being painful, spoil the beauty of the face. The following remedy was recommended in such cases – “If the rind of *Bel* fruit (Aegle marmelos Corr.) is levigated (i.e. powdered and mixed) in a woman's milk and the paste thus prepared is applied to the cracked lips, within 10 days the cracking will stop and the cracks will heal[5].”

### Skin lightning and exfoliating scrub

A fair skin has always been an attraction for Indians. The following paste used to be applied to the body to make the skin a shade or two lighter and give it a natural glow – “Pound together The root of *Kosta* [Kooth or Kushtha, English name: Costus.][6] (*Saussurea lappa Clarke*.), *Til* seeds (*Sessamum indicum* Linn.), the leaves of *Sirisa* (*Albizzia lebbek* Benth.), the leaves of *Chopda* (*Pongamia pinnata* Pierr.), the wood of *Devdar* (*Cedrus deodara* Roxb.) and the wood of *Zadali Haled* (*Berberis aristata* DC.) Roast this mixture between dried cakes of Buffalo dung, then take it out and grind it properly to a fine powder. If the paste made from this compound is applied to the body for three consecutive days, the above mentioned desired results will be obtained”.[5]

### Cure for dandruff

“Pound khas-khas seeds (*Papaver somniferum* Linn.) in milk and apply to the scalp. It will cure dandruff”.[5]

### Rejuvenation process (Kayakalpa)

A very famous rejuvenation treatment called *Kayakalpa* used to be practiced. The meaning of the word is to make a person look young, bring about a change in the color of the hair and texture of the skin, improve the eyesight and so forth. “Take equal quantities of *Kadunimba* (*Azadirachta indica* Juss.) leaves, *Maka* (*Eclipta alba* Haask.) leaves, *Mundi* [Gorakhmundi][7] (*Sphaeranthus indicus* Linn.) leaves, *Nirgundi* [Nagoda, Nirgundi and Nirgunda][8] (*Vitex negundo* Linn.)leaves and *Vova* (*Carum copticum* Benth.) leaves.

Dry all the 5 ingredients in the shade. Then powder this mixture. Take internally two pinches of this powder twice a day. While the subject is under this treatment, the diet should mainly consist of milk and rice only. Quite soon the person will look younger, the skin will become lustrous and even the grey hair will turn black”.[5]

### Depilatory

The presence of hair on arms, face, legs and pubic area, was considered an eyesore, and certain formulae were practiced to remove them.

“Pound together dried fruits of *Aavalakatti* (*Emblica officinalis* Gaertn.) and dried fruits of *Pimpali* (*Piper longum* Linn.). Soak this mixture in the milky latex of *Nivadunga* (Cactus: *Euphorbia nivulia* Ham.) If this compound is applied to the desired place, the hair from that area will fall off”.[5]

### Breast developers

“Powder together the root of *Aswagandha* (*Withania somnifera* Dunal.), the fruit of *Gajapimpali* (*Scindapsus officinalis* Schott.), the root of *Kosta* (*Saussurea lappa* Clarke.), and the rhizomes of *Vekhanda* [Vekhanda, English name: Sweet flag.][9] (*Acorus calamus* Linn.). To this powder add butter made from buffalo's milk and massage the bust with this medicated butter. This will increase the bustline and make it firm and shapely”.[5]

Some more formulae from other sources, earlier than 18^th^ and 19^th^ cent A.D. include the following:

### Face pack

“Take *Masura*—a lentil common in India (*Lens culinaris* Medic.) and pound with Madhu (Honey). The paste so prepared, rubbed for seven nights, gives the splendor of the petals of the white lotus flower to the face”.[10]

### Cure for pimples

“The application of plaster composed of *Kustumburu* [Dhana, Dhania, English name: Coriander][11] (*Coriandrum sativum* Linn.), *Vacha* or *Vekhanda* (*Acorus calamus* Linn.), *Lodhra* [Lodhar, Lodhra. English name: The Lodh tree][11] (*Symplocos racemosa* Roxb.) and *Kushtha* or *Kosta* (*Saussurea lappa* Clarke.) pasted together is also recommended for curing pimples”.[12,13]

**Figure 1:**
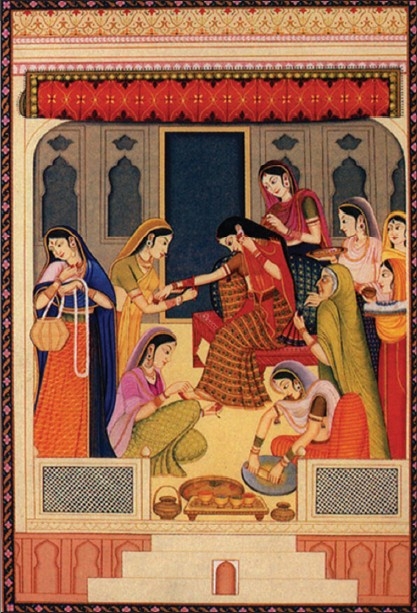
Artist's impression of use of cosmetics by women in ancient India from author’s book.[1]

### Mouth freshener

I quote a verse translated from Sanskrit: “Oh! beautiful damsel, make fragrant quickly, *Poog-phala* i.e. *Supari* or Betel nut (*Areca catechu* Linn.), for rulers of the earth (Kings) by mixing together *Kushtha* (*Saussurea lappa* Linn.), *Tagara* (*Valeriana wallichii* DC.), *Jatiphala* (*Myrstica fragrans* Houtt.), *Karpoora* (*Cinnamomum camphora* Nees and Eberm.), *Lavanga* (*Syzygium aromaticum* Merrill and Perry.) and *Ela* (*Ellettaria cardamomum* Maton)”.[14]

### Cure for lice and nits

“On tying the head with a piece of cloth dipped in the juice of *Phanivalli* (Piper betel Linn.) or ‘*Paan*” leaves, to which has been added *Paratda* (Mercury), lice and nits would be finished off.[15]

### General hair remedy and cure for the prematurely graying

“Juice of *Bhringaraja* or *Maka* (*Eclipta alba* Hassk.), together with Lohakitta (Iron-rust: Non-botanical. Iron oxide, normally Red oxide.), *Phalatrikam* or *Triphala* = Collection of three fruits, *viz*. *Harada* [Hirda, English name: *Chebulic myrobalan*][16] (*Terminalia chebula* retz.), *Beheda* [Behada, English name: *Belleric myrobalan*][6] (*Terminalia bellerica* retz.) and *Avala* [Amla, English name: *Emblic myrobalan*][17] (*Phyllanthus emblica* Gaertn.), cooked in oil when applied (to the scalp), would cure dandruff, itching, alopecia and would also darken the hair, which have become grey prematurely.[18]

### Deodorant powder

“The powder from the barks of *Sahakara* [*Aam, Amba*, English name: Mango][17] (*Mangifera indica* Linn.) tree and *Dadima* [*Dadim, Dalimba*; English name: Pomegranate][17] (*Punica granatum* Linn.) tree, mixed with *Shankha* (Fragrant Shell) powder and applied to the relevant part of the body, removes bad odour. The powder made of *Chincha* (*Tamarindus indica* Linn.) and *Karanja* (*Pongamia glabra* Vent.) seeds, if applied also removes bad odour.[19,20]

Examples of above given formulae serve to give some idea of Ancient Indian researchers and their contribution to Indian Medical Lore.

The research involved in this work was to compile the cosmetic formulae from various sources and then to find the proper equivalent botanical names for the Sanskrit and *Prakrit* terminologies (For the ingredients used in the formulae). This was done with the help of native people and experienced vaidyas in South India and Maharashtra. Identification was done by studying the properties of the plant and if these plant names were the actual ingredients used.

Some of the formulae are still in use by some rural women in the interiors of India. Some formulae are in use even today by vaidyas practicing the Ayurveda branch of medicine.

This study forms mainly an ethnobotanical contribution to our knowledge and is hoped that it forms the basis for further chemical, clinical and allied investigations in the cosmetic and therapeutic aspects of the Indian botanicals.
